# Pump‐Less Platform Enables Long‐Term Recirculating Perfusion of 3D Printed Tubular Tissues

**DOI:** 10.1002/adhm.202300423

**Published:** 2023-09-19

**Authors:** Feng Zhang, Dawn S. Y. Lin, Shravanthi Rajasekar, Alexander Sotra, Boyang Zhang

**Affiliations:** ^1^ School of Biomedical Engineering McMaster University Hamilton ON L8S 4L8 Canada; ^2^ Department of Chemical Engineering McMaster University Hamilton ON L8S 4L8 Canada

**Keywords:** blood vessels, kidney proximal tubule, monocytes, organ‐on‐a‐chip, unidirectional flows

## Abstract

The direction and pattern of fluid flow affect vascular structure and function, in which vessel‐lining endothelial cells exhibit variable cellular morphologies and vessel remodeling by mechanosensing. To recapitulate this microenvironment, some approaches have been reported to successfully apply unidirectional flow on endothelial cells in organ‐on‐a‐chip systems. However, these platforms encounter drawbacks such as the dependency on pumps or confinement to closed microfluidic channels. These constraints impede their synergy with advanced biofabrication techniques like 3D bioprinting, thereby curtailing the potential to introduce greater complexity into engineered tissues. Herein, a pumpless recirculating platform (UniPlate) that enables unidirectional media recirculation through 3D printed tubular tissues, is demonstrated.The device is made of polystyrene via injection molding in combination with 3D printed sacrifical gelatin templates. Tubular blood vessels with unidirectional perfusion are firstly engineered. Then the design is expanded to incorporate duo‐recirculating flow for culturing vascularized renal proximal tubules with glucose reabsorption function. In addition to media recirculation, human monocyte recirculation in engineered blood vessels is also demonstrated for over 24 h, with minimal loss of cells, cell viability, and inflammatory activation. UniPlate can be a valuable tool to more precisely control the cellular microenvironment of organ‐on‐a‐chip systems for drug discovery.

## Graphical abstract

A pumpless recirculating platform (UniPlate) that enables unidirectional media recirculation through 3D printed tubular tissues  is developed. Using this platform, the formation of complex multilayered blood vessels and vascularized renal proximal tubule tissues is demonstrated. This platform can also recirculate immune cells for at least 24 h with minimal activation, making it suitable for investigating immunological surveillance in organ‐on‐chip systems.

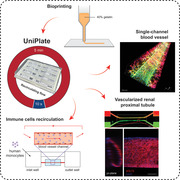

## 1 Introduction

Blood vessels are routinely exposed to dynamic mechanical stimuli, including shear stress exerted by blood flow and cyclic strain from pulsatile pressure generated by cardiac contraction. Blood flow remains a significant factor in regulating the structure and function of vascular tissues.^[^ ^1^ ^]^ For instance, in the presence of luminal shear stress, endothelial cells tend to stay quiescent and form tighter barriers, while vessel sprouting is activated when the interstitial flow is predominant.^[^ ^2^ ^]^ The permeability of blood–brain barriers was found to decrease when the vessels are exposed to pulsatile pressure.^[^ ^3^ ^]^ In addition to blood vessels, fluid flow also plays an important role in regulating tubular epithelial tissues. In renal proximal tubules, luminal perfusion was reported to facilitate the formation of microvilli brush border, which is a key cellular apparatus for sensing fluid flow.^[^ ^4^ ^]^ High fluid shear stress has been shown to accelerate cell differentiation and apoptosis at the tips of intestinal villi in 3D small intestinal models, thereby reproducing the cell turnover process on native intestinal.^[^ ^5^ ^]^

In addition to the presence of flow, the specific direction of flow has also been documented to affect cell morphology and inflammatory signaling activation in endothelial cells.^[^ ^6^, ^7^ ^]^ Under unidirectionalperfusion, endothelial cells preferentially display an elongated morphology and align in the flow direction compared to the bidirectional flow condition.^[^ ^8^ ^]^ Unidirectional flow is also beneficial for reducing mitochondrial reactive oxygen species production and protecting the mitochondrial DNA integrity of endothelial cells.^[^ ^9^ ^]^ Anatomically, blood flow in the human body is unidirectional and recirculatory. Nutrient‐rich blood is transported and distributed to all tissues by arteries, and blood with metabolic byproducts is returned to the heart via the veins. The liver lobule has a specific hierarchical anatomy with blood flowing from the hepatic artery to the portal vein unidirectionally, which leads to the formation of multiple gradients, such as nutrients, oxygen, and hormones.^[^ ^10^ ^]^ Under the stimulation of unidirectional flow, vascularization of kidney organoids was enhanced with increasing vessel coverage, vessel junctional density, and vessel length, which further promoted kidney organoids maturation.^[^ ^11^ ^]^ On the contrary, bidirectional flow is found to preferentially present in regions of atherosclerotic lesions with activated endothelial nitric oxide synthase signaling, which is a high‐risk factor in many cardiovascular events related to endothelial dysfunction and atherosclerosis.^[^ ^12^, ^13^ ^]^ Therefore, it is necessary to consider flow direction and pattern when engineering vascular tissue models.

Existing approaches established to realize unidirectional flow in organ‐on‐chip models tend to use a syringe or peristaltic pump to perfuse and circulate media.^[^ ^14^, ^15^, ^16^ ^]^ However, bulky supporting systems, tedious tubing connections, the risk of trapping bubbles, and the presence of dead volumes in tubing often limit the usability and scalability of such systems. To overcome problems derived from pumps and tubing, gravity‐driven pumpless organ‐on‐a‐chip devices with unidirectional flow are attractive alternatives.^[^ ^17^, ^18^, ^19^ ^]^ The basic principle is to introduce a bypass channel connecting the inlet and outlet reservoirs for medium recirculation.^[^ ^20^ ^]^ With the assistance of a rotating platform, unidirectional flow can be achieved.^[^ ^21^ ^]^ Moreover, by elaborately designing connections between the reservoirs, Wang et al. demonstrated a microfluidic Unichip device to obtain continuous unidirectional flow on a rocking system, eliminating stagnation flow or backflow in recirculation.^[^ ^22^ ^]^ However, these existing platforms are generally limited to closed microfluidic channels/chambers with limited structural complexity. Closed microfluidic systems are usually polydimethylsiloxane (PDMS) ‐based and do not integrate well with techniques, such as 3D bioprinting, to produce more complex tissue structures. Moreover, immune cell circulation is yet to be demonstrated in gravity‐driven perfusion platforms with recirculating flow. Therefore, a pumpless recirculating system that supports 3D bioprinted tissues and recirculation of immune cells in an open‐well format is much needed.

In this study, we developed a pumpless recirculating platform that enabled unidirectional fluid flow through 3D printed vascular/tubular tissues. Unidirectional flow and recirculation were controlled by a rocker with a programmable dwell time at different tilt angles. We demonstrated for the first time the formation of 3D tubular blood vessels with different geometries, complex tubular blood vessels surrounded by fibroblasts and pericytes, and vascularized renal proximal tubules with active glucose transport functions under a recirculating flow driven by gravity. This platform can also recirculate immune cells for at least 24 h with minimal activation, making it suitable for investigating immunological surveillance in diseased blood vessels and distal tissues.

## 2 Results

### 2.1 UniPlate were Fabricated with Injection Molding and 3D Printing

**Figure 1:**
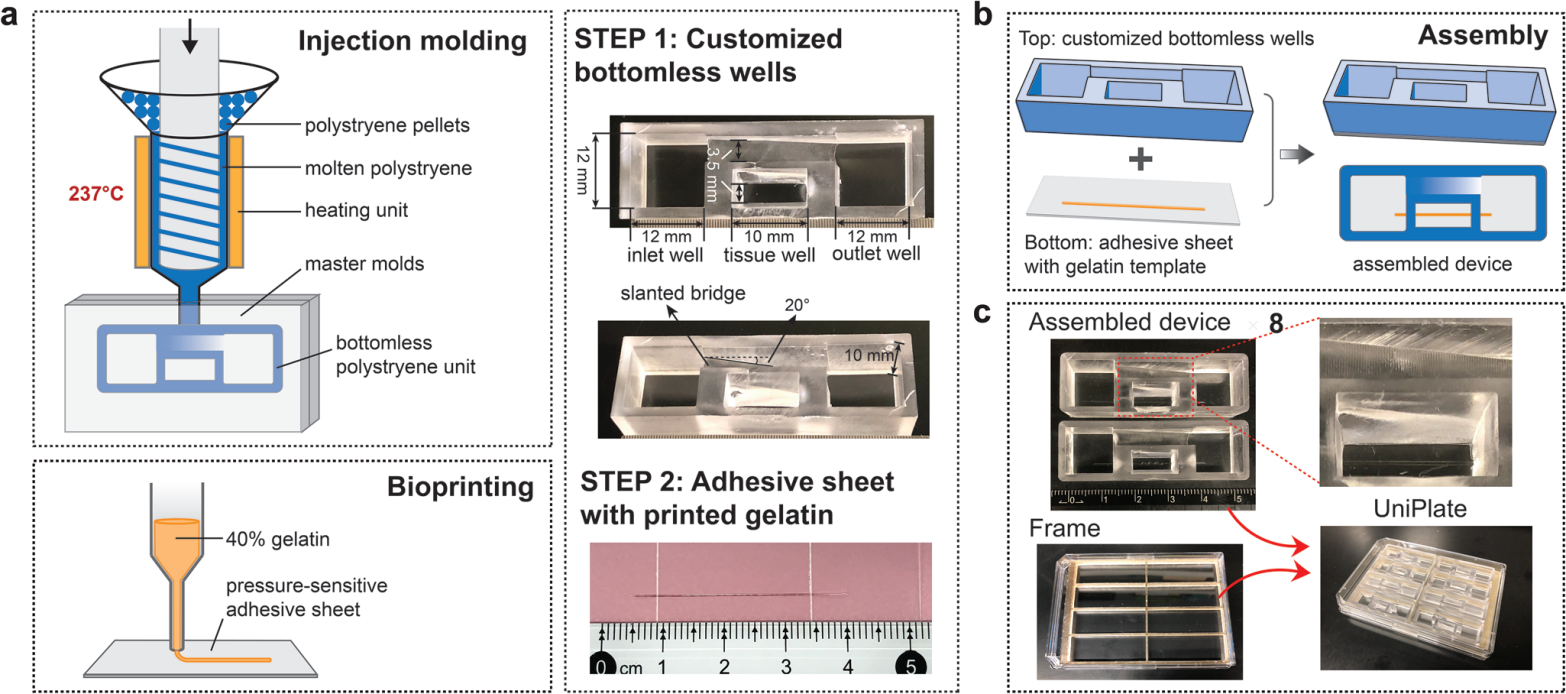
Fabrication and setup of UniPlate. a) Schematic illustration and image of the UniPlate fabrication process, including manufacturing of customized bottomless wells (STEP 1) and gelatin printing on pressure‐sensitive adhesive sheets (STEP 2). b) Schematic of the device assembly. c) Image of assembled UniPlate. Each plate contained eight devices grouped together using a 3D printed frame that fits inside a OneWell Plate. The inset shows the tissue well with 3D printed gelatin sacrificial template.

**Figure 2:**
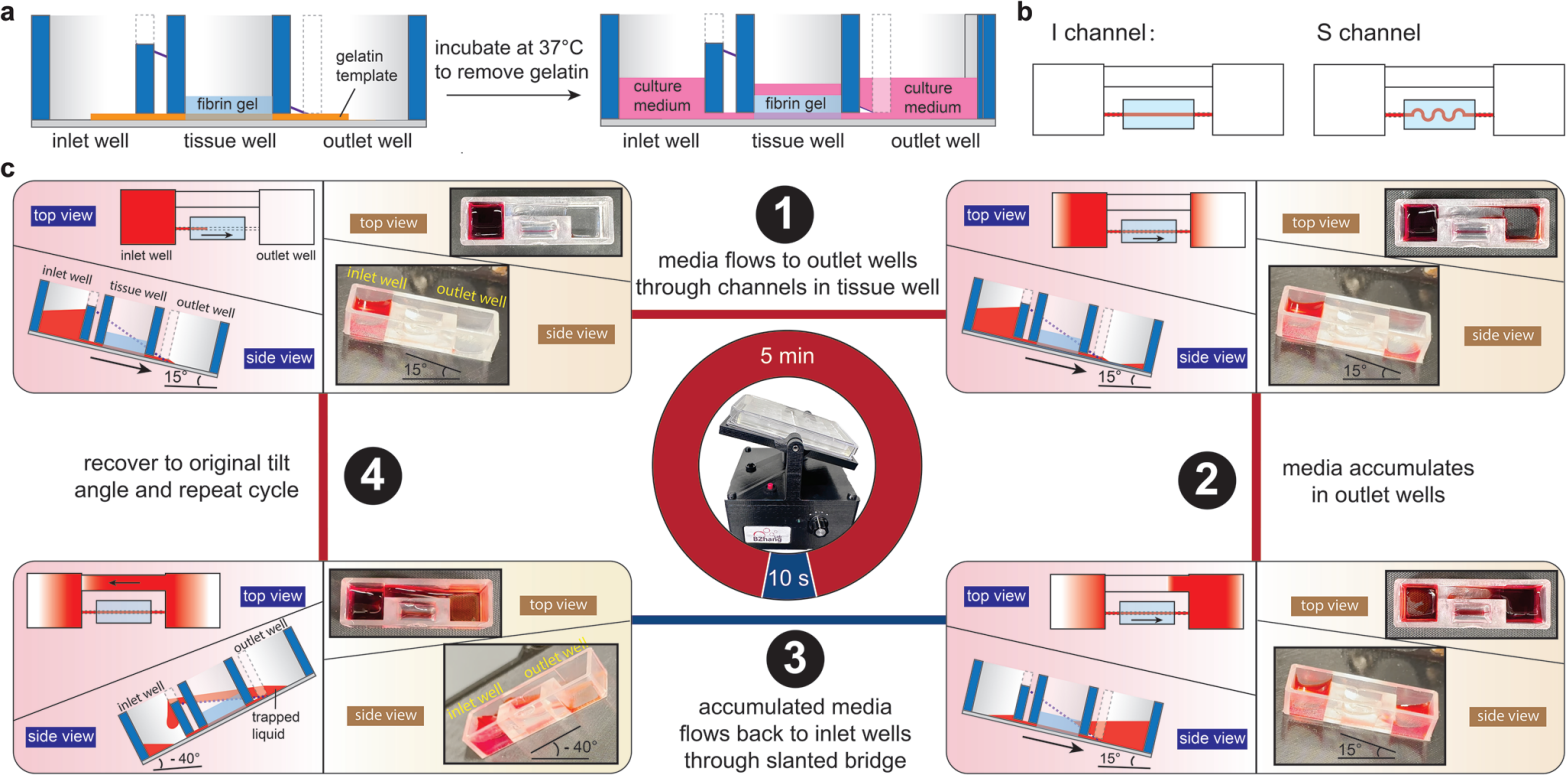
Operation and recirculating flow in UniPlate. a) Schematic illustration of gelatin removal and perfusable channel formation after casting fibrin gel in the tissue wells. b) Single‐channel UniPlate in two different designs: linear (I channel) and tortuous (S channel). c) Schematic illustration and time‐lapse photos of a complete perfusion cycle. Red food dye was added to the inlet well to better visualize the process.

We developed a pumpless recirculating platform in the form of a standard 24‐well plate that supports recirculating unidirectional perfusion of 3D printed vascular/tubular tissues on a programmable rocking system. The platform is modular and partially injection‐molded from polystyrene, which is the industry‐standard material for microtiter plates, thus minimizing the nonselective drug absorption issues associated with traditional PDMS‐microfluidic devices.^[^ ^23^ ^]^ Each UniPlate contains eight fully assembled devices secured on a customized frame. Each device was made of two components: a customized bottomless well and a thin pressure‐sensitive adhesive sheet with 3D printed gelatin sacrificial template. The device was fabricated using injection molding and 3D printing (**Figure** **1**a). Customized bottomless wells made of polystyrene were manufactured by injection molding, and bioprinting was used to produce the gelatin template on pressure‐sensitive adhesive sheets. The assembled device contained three separate wells (inlet, tissue, and outlet wells) and a slanted bridge that is directly connected to the inlet and outlet wells (Figure 1b). The slanted bridge was designed to slant at 20° angle. The three wells were connected by two microchannels (400 × 400 µm^2^) (Figure S1, Supporting Information). These channels serve as inlets and outlets to the tissue well where the 3D printed tissues will be contained. One UniPlate can hold eight devices independently removable from the plate for analysis (Figure 1c). To produce perfusable tissues, a subtractive manufacturing method was adopted with some modifications.^[^ ^24^ ^]^ Specifically, gelatin was selected as the sacrificial material and could be printed with different patterns on the pressure‐sensitive adhesive sheet using an extrusion‐based bioprinter. Pressure‐sensitive adhesive (PSA) was used because of its great biocompatibility, and it shows minimal absorption to small hydrophobic drugs comparing to PDMS (Figure S2, Supporting Information).^[^ ^25^ ^]^ The device was then assembled by hand‐pressing the bottomless wells with pressure‐sensitive adhesive sheets containing 3D printed gelatin. Parts of the printed gelatin template were designed to align with the inlet and outlet channels after assembly to establish flow connections. To produce the tissues, fibrin gel was cast over the gelatin template inside the tissue wells (**Figure** **2**a). After gelation, gelatin can be dissolved at 37 °C, leaving an open channel that can be populated with endothelial or epithelial cells. The sacrificial manufacturing method using gelatin as the scaffold showed high reproducibility in building simple single‐channel tubular tissues (Figure S3, Supporting Information). Native blood vessels and epithelial tubules have diverse geometries. For example, the uterine spiral artery and proximal tubule vessels are curved and tortuous.^[^ ^4^, ^26^ ^]^ To demonstrate the flexibility offered by the extrusion printing process in tissue customization, we produced two designs to model vessels with linear and tortuous shapes, termed I and S channels, respectively (Figure 2b).

For unidirectional perfusion and recirculation, the plate was placed on a programmable rocker (Figure 4a, Supporting Information). When the rocker was tilted at a positive angle (e.g., 15°), the media level in the inlet well was higher than that in the outlet wells; thus, the media flowed through the tubular channel in the fibrin gel to the outlet wells (Figure 2c). The media can only perfuse through channels embedded inside the fibrin gel and accumulate slowly in outlet wells because the slanted bridge allows only one‐way perfusion from the outlet to inlet wells. After 5 min of forward perfusion, the rocker stage was flipped to a negative tilt angle (e.g., −40°) to allow the accumulated media in the outlet wells to flow back to the inlet wells immediately via the slanted bridge, which offers a much lower flow resistance than the tubular channel inside the fibrin gel. The rocker held this tilt position for only 10 s, which is more than sufficient to allow media recirculation. Finally, the rocker changed the tilt direction again and recovered to the original positive tilt angle to start a new cycle. Red food dye was added to the inlet wells to better visualize a complete perfusion cycle in UniPlate (Video S1, Supporting Information). Therefore, the flow generated in Uniplate is not entirely unidirectional. The flow is pseudo‐unidirectional flow with a brief backflow when the rocker tilted at a negative angle during the recirculation.

### 2.2 Rocker Tilt Angle Regulates Flow Rate and Residual Volume in UniPlate

**Figure 3:**
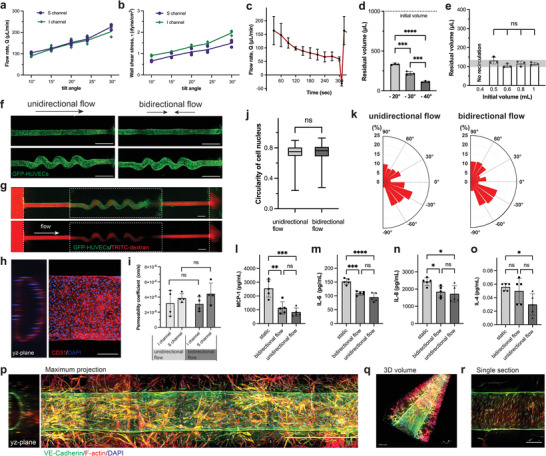
Flow characterization and unidirectional perfusion of 3D tubular vessels in a UniPlate. a,b) Correlations of flow rate and wall shear stress with rocker perfusion tilt angles; *n* = 3 devices for each condition. c) Flow rate changes in printed single‐channel tubule in a complete perfusion cycle. The flow rate for forward perfusion was quantitatively measured every 30 s. *n* = 4 devices for each condition. d) Correlation of residual volume in UniPlate outlet wells to recirculating tilt angles when the initial volume of media added to the outlet wells is 1 mL; *n* = 3 devices for each tilt angle condition. Statistical significance was determined using one‐way ANOVA with the Holm–Sidak method. ***p < 0.001, ****p < 0.0001. e) Correlation of residual volume in UniPlate with initial media volume in outlet wells at −40° recirculating tilt angle; *n* = 3 devices for each condition. Statistical significance was determined using one‐way ANOVA. “ns” indicates not statistically significant. f) Fluorescent images of blood vessels formed after 7 days of culture under unidirectional and bidirectional flow; scale bar:1 mm. g) Fluorescence images of a perfused (65 kDa dextran, red) blood vessel formed under unidirectional flow for permeability quantification on day 8; scale bar:1 mm. h) Confocal projected z‐stack fluorescence images of vessels with unidirectional flow in UniPlate. The cells were stained with CD31 (red) and DAPI (blue). Scale bar: 200 µm. i) Permeability coefficient quantification of vessels under unidirectional and bidirectional flow culture conditions; *n* = 3 vessels per group. Statistical significance was determined using Kruskal–Wallis one‐way ANOVA on ranks. “ns” indicates not statistically significant. j,k) Cell nucleus morphology j) and nucleus orientation k) quantification of endothelial cells in vessels after 7 days of culture with unidirectional or bidirectional flow; *n* = 3 vessels analyzed for each culture condition. Statistical significance was determined using Kruskal–Wallis one‐way ANOVA on ranks. “ns” indicates not statistically significant. To quantify the cell nucleus orientation, 214 cells from three vessels were counted in the unidirectional group, and 238 cells from three vessels were counted in the bidirectional group. 0° indicates the cell nucleus orientate along the flow direction. l–o) Quantification of inflammation‐related cytokines secretion levels, including l) MCP‐1, m) IL‐6, n) IL‐8, and o) IL‐4 in collected media perfusates from blood vessels cultured under static, bidirectional flow and unidirectional flow conditions. *n* = 5 for each condition. Statistical significance was determined using one‐way ANOVA with the Holm–Sidak method. *p < 0.05, **p < 0.01, ***p < 0.001, ****p < 0.0001, “ns” indicates not statistically significant. p) Stitched confocal maximum projected z‐stack fluorescence images of a blood vessel with fibroblasts and pericytes embedded in fibrin gel under unidirectional flow. The cells were stained with VE‐Cadherin (green), F‐actin (red), and DAPI (blue). Scale bar: 1 mm. q) 3D‐reconstituted confocal fluorescence image of the blood vessel. r) Confocal fluorescence image of blood vessel tissue at a single section. Scale bar: 200 µm.

The flow rates in the single‐channel design were evaluated by collecting and measuring the volume of media perfusate from the inlet to the outlet. The flow rates can be adjusted from 100 to 200 µL min^−1^ by increasing the tilt angle (**Figure** **3**a). The calculated wall shear stress within the tubular blood vessels (with a diameter of 500–700 µm) varies from 0.7 to 2 dyne cm^−2^ when the tilt angle changes from 10° to 30° (Figure 3b), which is comparable with that of venous in vivo (with the diameter of 0.03–12.5 mm and shear stress of 1–4 dyne cm^−2^).^[^ ^27^ ^]^ Importantly, the size of printed tubule is adjustable by changing the needle size used in bioprinting. The resolution of extrusion‐based bioprinting was reported to range from 100 to 500 µm.^[^ ^28^ ^]^ To understand the flow rate changes during perfusion, we evaluated the flow rate every 30 s, which showed that the flow rate decreased gradually as the liquid level in inlet wells reduced (Figure 3c). With the connecting bridge, the majority of media in outlet wells could flow back to inlet wells immediately. However, some media was retained in the outlet wells. To maximize recirculation, we evaluated the residual media volume in the outlet wells at different recirculation tilt angles. The minimum residual media volume was achieved at −40° tilt angle, which is the largest accessible tilt angle for the rocker. Approximately 100 µL of media, 10% of the initial volume, were not recirculated (Figure 3d). Changing the initial media volume in the outlet well did not affect the volume of residual media (Figure 3e; and Figure S4b, Supporting Information). It is important to note that the residual media will mix with the media from the next perfusion cycle and eventually circulate back to the inlet wells. However, to achieve the highest recirculation efficiency, −40° tilt angle was chosen as the recirculation tilt angle, whereas the perfusion tilt angle was set to 15°.

### 2.3 Tubular Blood Vessels can be Perfused Recircularly in UniPlate

To produce a blood vessel model with unidirectional flow, GFP‐expressing human umbilical vein endothelial cells (GFP‐HUVECs) were seeded in tubular channels embedded in fibrin gel. After 7 days of culture, endothelial cells proliferated and formed a confluent lumen under both unidirectional and bidirectional flow conditions (Figure 3f,g). Engineered blood vessels showed an open lumen surrounded by endothelial cells (Figure 3h; and Figure S4c, Supporting Information). To evaluate the vessel permeability, TRITC‐conjugated dextran (65 kDa) was added to the inlet wells. The blood vessels formed under unidirectional flow showed a permeability of 3.5 × 10^−6^ cm s^−1^, which is comparable to those of native blood vessels (0.15 × 10^−6^ cm s^−1^) and other reported blood vessel chip models (4.1 × 10^−6^ cm s^−1^) (Figure 3i).^[^ ^29^, ^30^ ^]^ The permeabilities of the blood vessels under unidirectional and bidirectional flows were also compared and surprisingly showed no significant difference under our current experimental condition.

Cell nuclei elongation was evaluated by comparing nuclei circularity and orientation of endothelial cells in blood vessels cultured under unidirectional and bidirectional flow conditions. However, there was no significant difference in cell nuclei circularity under unidirectional and bidirectional conditions (Figure 3j), indicating that endothelial cells in 3D tubular blood vessels do not show apparent cell elongation under unidirectional or bidirectional flow under shear stress at ≈1 dyne cm^−2^. There was also no significant difference in cell nucleus orientation between unidirectional and bidirectional conditions (Figure 3k). Endothelial cells tended to orientate at an angle ranging from −75° to −15° to the flow direction in both conditions. Moreover, we compared secretion levels of inflammatory‐related cytokines in media perfusates from blood vessels cultured under static, bidirectional flow, and unidirectional flow conditions (Figure 3l–n; and Figure S5, Supporting Information). The secretion levels of MCP‐1, IL‐6, and IL‐8, which are biomarkers involved in promoting, activating, or accelerating the coagulation cascade and lesion formation in atherosclerosis, displayed a slight decreasing tendency in the unidirectional group compared with the bidirectional condition.^[^ ^31^, ^32^, ^33^ ^]^ MCP‐1, IL‐6, and IL‐8, also exhibited significantly higher secretion levels in statically cultured blood vessels compared to bidirectional and unidirectional flow groups (Figure 3l–n). This suggests early acute inflammation in static blood vessels, further confirming the importance of flow in engineering functional blood vessels. More interestingly, IL‐4, which was reported to be related to the development of atherosclerosis, showed a significant reduction in the unidirectional flow condition compared to the static condition, but this result was not observed in the bidirectional group (Figure 3o).^[^ ^34^ ^]^

Unlike the 2D culture condition on closed PDMS/PMMA (polymethyl methacrylate) ‐based devices, we found that endothelial cells in 3D perfusable vascular tissues responding to unidirectional flow did not show significant differences in cell nuclei elongation and permeability compared to the bidirectional flow condition at the cellular level. These results could be explained by the differences in matrix stiffness between the fibrin gel (1–8 kPa) and substrates used to support 2D endothelial monolayer cultures (0.8–10 MPa for PDMS) in prior studies.^[^ ^35^, ^36^, ^37^ ^]^ Shear stress could also be a contributing factor for the difference in observation. In a previous study, the enclosed PDMS/PMMA devices had a higher shear stress in the range of 5–10 dyne cm^−2^ which could promote endothelial cell alignment.^[^ ^38^ ^]^ Furthermore, mechanical stimuli in 3D blood vessels are much more complex than those in 2D endothelium. In 3D, the endothelium was also exposed to transendothelial interstitial flow into the gel, which could have an impact on the cells. The underlying matrix could also be sufficiently soft to deform under flow.^[^ ^39^ ^]^ The impact of these additional mechanical factors on cellular morphology must also be considered in this 3D model. For instance, we noticed that vessel sprouting could be induced in our model by adjusting the dwell time of the rocker from 5 to 15 min (Figure S6, Supporting Information). Based on the current flow rate, it takes about 8 min for 1 mL of culture media to flow into the outlet well. In the 15 min settings, as the media drains from the inlet wells, the flow rate and fluid shear stress will reduce significantly over time, which makes transendothelial interstitial flow from the interstitial space more dominant over time. Previous studies have demonstrated that interstitial flow can induce vessel sprouting, which is consistent with our observations.^[^ ^2^ ^]^ Therefore, the 5 min setting helped to maintain continuous luminal flow, which suppresses vascular sprouting. Despite of optimizing the rocker setting, the pseudo‐unidirectional flow condition in our work might still be different from continuous unidirectional flow condition to some extent. During the 10 s of recirculation, endothelial cells in 3D tubular tissues are exposed to a brief backflow, which could potentially affect cell morphology in response to unidirectional perfusion. Further investigation is needed in the future. Complex multilayered tissue structures can also be developed in UniPlate, by embedding stromal cells, spheroids, or organoids in the gel matrix. As a proof‐of‐concept, we embedded primary fibroblasts and pericytes in fibrin gel around the endothelialized channel to mimic cellular compositions of capillary‐like blood vessels in vivo. The blood vessel displayed an open lumen tubular structure formed by confluent endothelial layer, which is further surrounded by stromal cells in close proximity (Figure 3p,q). Different from the condition without stromal cells, endothelial cells demonstrated a highly elongated morphology and spiral around the tubular channel along the flow direction (Figure 3r).

### 2.4 Vascularized Renal Proximal Tubule with Recirculating Flow Demonstrated Glucose Reabsorption Function

**Figure 4:**
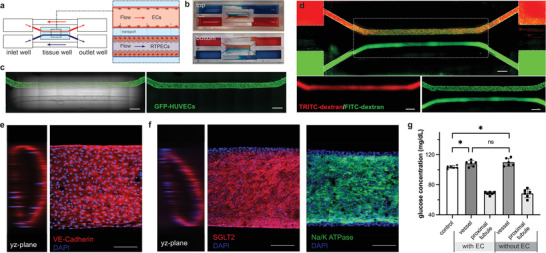
A vascularized proximal tubule with recirculating flow can reabsorb glucose in UniPlate. a) Schematic of dual‐channel recirculation flow setup. b) Top and bottom views of a 3D vascularized proximal tubule tissue perfused with blue and red food dyes. c) Images of a proximal tubule next to a tubular blood vessel (green) on day 4 after cell seeding; scale bar:1 mm. d) Fluorescence images of dextran‐perfused (65 kDa, red and green) vascularized proximal tubule tissue; scale bar:1 mm. e,f) Confocal fluorescence images of vascularized proximal tubule tissue stained for VE‐cadherin (red), SGLT2 (red), Na/K ATPase (green), and DAPI (blue). Scale bar: 200 µm. g) Quantification of glucose concentration in media perfusates from vascular and tubular channels on day 7 after reaching confluency. *n* = 6 for each condition. Statistical significance was determined using one‐way ANOVA with the Holm–Sidak method. *p < 0.05; “ns” indicates not statistically significant.

Next, we expanded the single‐channel UniPlate design to incorporate two individually perfusable networks inside the same gel matrix to reproduce a vascular–tubular network that emulated vascularized proximal tubule complexes in the kidney (**Figure** **4**a). The two independent channels were perfused with two sets of inlet and outlet wells, and two slanted bridges for recirculation. Similar to single‐channel blood vessel, flow rate in both vascular, and tubular channels decreased over time. The diameter of printed renal proximal tubule is from 550 to 700 µm (Figure S7, Supporting Information), which is comparable with that of other published works mimicking renal proximal tubules.^[^ ^4^, ^40^ ^]^ Due to the resolution constraints of extrusion‐based bioprinting, the bioprinted tubule tissues are relatively larger than the in vivo proximal tubule, which has an outer diameter of 70 µm.^[^ ^41^ ^]^ However, the size differences may not really impact the function of the kidney proximal tubule, as the glucose reabsorption of engineered proximal tubule tissues with the diameter varying from 120 to 700 µm has been demonstrated in this work and other published works.^[^ ^42^ ^]^ Nevertheless, smaller tubule structures could be established by controlling parameters in 3D printing. Blood vessels and proximal tubule tissues were produced by seeding endothelial and renal proximal tubule epithelial cells into their corresponding channels. The media were perfused unidirectionally through both the blood vessels and proximal tubule and visualized with red and blue dyes (Figure 4b). We found that vascular and proximal tubule networks formed confluent tubular structures after 4 days of culture (Figure 4c), and both blood vessels and proximal tubule networks demonstrated a high barrier function that confines large proteins (Figure 4d). Similar to the single‐channel tubular vessel, a 3D luminal structure of the vessel and proximal tubule was observed. VE‐cadherin staining of the blood vessels (Figure 4e) showed continuous intercellular junctions, indicating the formation of a tight endothelial barrier.^[^ ^43^ ^]^

Glucose reabsorption is one of the primary functions of the renal proximal tubule, which transports glucose from the proximal tubule to the neighboring blood vessels. In this process, sodium‐glucose cotransporters are involved in the assistance of Na/K ATPase to maintain osmotic equilibrium and membrane potential in cells.^[^ ^44^ ^]^ The proximal tubule contains the glucose transporters SGLT2 and Na/K ATPase (Figure 4f). To model glucose transport, both the blood vessel and proximal tubule were treated with a medium containing ≈100 mg dL^−1^ glucose, which is in the range of normal blood glucose levels for nondiabetics. After 24 h of unidirectional flow, the glucose levels in perfusates from each channel were measured. Compared to the initial concentration of glucose in fresh media, perfusates from the blood vessels showed an increase in glucose concentration with reduced glucose levels in the proximal tubule (Figure 4g), indicating the active transport of glucose from the proximal tubule to blood vessels. Moreover, 3D vascularized proximal tubule tissues with or without endothelial cells exhibit similar glucose levels in the vessel perfusates, indicating that the proximal tubule is the dominant factor in reabsorbing glucose, while the blood vessel passively takes up glucose from the interstitial space, which is consistent with previously published studies.^[^ ^40^ ^]^

### 2.5 Immune Cells Can Recirculate in UniPlate

**Figure 5:**
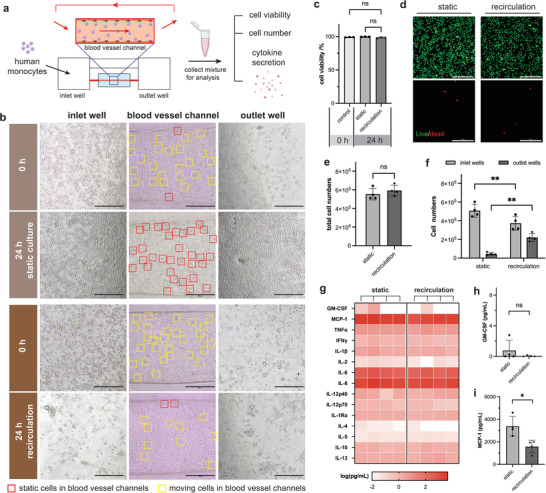
UniPlate enables long‐term recirculation of monocytes without pro‐inflammatory activation. a) Schematic of monocyte (THP‐1) recirculation in UniPlate and analysis. b) Time‐lapse images of monocytes at the bottom of the inlet and outlet wells and inside the blood vessel channels after 24 h of static and recirculation cultures. Static and moving cells in the blood vessel channels are labeled with red and yellow squares, respectively. Scale bar: 200 µm. c) Quantification of cell viability before (0 h) and after 24 h of static or recirculation culture in UniPlate; *n* = 3 for each condition, and cell viability at 0 h was used as the control. Statistical significance was determined using one‐way ANOVA. “ns” indicates not statistically significant. d) Representative live/dead staining images of monocytes after 24 h of static or recirculating culture. Scale bar: 200 µm. e,f) Quantification of e) total monocyte cell number and f) monocyte cell numbers in inlet and outlet wells collected from media perfusates after 24 h of static culture or recirculation. *n* = 3 for each condition. Statistical significance was determined using one‐way ANOVA with the Holm–Sidak method. **p < 0.01, “ns” indicates not statistically significant. g) Quantification of cytokine secretion levels, including monocyte modulatory cytokines h) GM‐CSF and i) MCP‐1, in collected media perfusates after 24 h of static and recirculating cultures. *n* = 4 for each condition. Statistical significance was determined using one‐way ANOVA with the Holm–Sidak method. *p < 0.05, “ns” indicates not statistically significant.

Blood is a multicomponent mixture containing fluids, plasma, blood cells, and platelets, and is an important part of any physiological model that involves blood vessels. The long‐term recirculation of whole blood or circulating immune cells in engineered blood vessel models using gravity‐driven flow remains challenging because of cell sedimentation issues in long‐term cultures. Cell settlement and aggregation occur when the cells are cultured under relatively static conditions with minimal convective mixing. However, with excessive agitation, inflammatory activation of circulating immune cells may also occur owing to mechanically induced stress. To investigate the feasibility of using UniPlate for recirculating immune cells, human monocytes (THP‐1) were recirculated for 24 h on the platform (Videos S2 and S3, Supporting Information). Cell number and viability were evaluated by live/dead staining, and media perfusates were collected for cytokine analysis (**Figure** **5**a). Under static conditions, as expected, most circulating monocytes were found to move in the vessel channel at 0 h but completely settled to the bottom of the inlet/outlet wells after 24 h of static culture (Figure 5b). The cells left inside the blood vessels were also found to be static. Compared to the static culture group, no apparent difference was detected in the recirculation group at 0 h, and all cells moved from the inlet well to the outlet well through the vessel channel. However, after 24 h of recirculation, many cells continued to flow inside the blood vessel, and cells in the inlet and outlet wells remained suspended in the medium with good dispersion. These results demonstrate the feasibility of monocyte recirculation in UniPlate. Furthermore, after 24 h of recirculation, the viability of monocytes was comparable to that of cells at 0 h (Figure 5c,d). The overall cell numbers in the perfusates collected from the static and recirculation groups were comparable, indicating no significant cell loss during recirculation (Figure 5e). However, a more even cell distribution in the inlet and outlet wells was observed in the recirculation group, with an increased cell number in the outlet wells and a reduced cell number in the inlet wells, indicating that monocytes were successfully perfused, recirculated, and continuously mixed in the medium throughout the entire device (Figure 5f). To investigate whether circulating monocytes were activated by mechanical stress during recirculation, we analyzed cytokine secretion. As expected, we did not observe any significant changes in the secretion levels of the inflammatory cytokines (Figure 5g). Specifically, GM‐CSF and MCP‐1 are key cytokines responsible for recruiting circulating monocytes to sites of inflammation. Both static and recirculation groups showed similar secretion levels of GM‐CSF lower than 0.6 pg mL^−1^ (Figure 5h). Interestingly, the secretion of MCP‐1 (Figure 5i) was significantly higher under the static condition than that in the recirculation condition, indicating that there was a certain level of inflammation in the culture, likely from endothelial cells, after 24 h of static culture. There was no significant difference in the secretion levels of other key pro‐inflammatory cytokines such as IL‐6, IL‐8, TNF‐α, and IFN‐γ (Figure S8, Supporting Information), indicating that monocytes were not significantly activated by recirculating flow. Overall, these results indicate that UniPlate supports monocyte recirculation.

## 3 Discussion

Microphysiological systems aim to recapitulate the key features of human tissue functions by precisely controlling how individual cells interact with each other and the surrounding microenvironment. Among the many different types of interactions between cells and their microenvironments, dynamic fluid flow is often an important factor affecting cellular behavior and function.^[^ ^45^, ^46^ ^]^ In this study, we demonstrated a pumpless recirculating platform that can produce a long‐term recirculating unidirectional flow of culture media and circulating immune cells in 3D tubular tissues, such as blood vessels and renal proximal tubules, embedded in fibrin gels. Here, we achieved unidirectional flow with a programmable rocker and customized well‐plate design. This gravity‐driven perfusion approach eliminates the issues of tubing connectivity, air bubbles, and dead volume in conventional microfluidic fluid control systems. Platforms that adopt gravity‐driven flows are highly scalable. Each plate can contain multiple devices and can be stacked on the rocker to increase the experimental throughput. The dimensions and layout of the UniPlate were designed to match those of standard commercial 24‐well plates, making it compatible with commercial plate readers and standard microscope imaging setups. We selected a 24‐well plate format owing to the physical constraints of our benchtop injection molder. However, in future work, based on the same design principle, the platform can be scaled up to a 384‐well plate format to further increase the experimental throughput and reduce the reagent usage per tissue.

The fabrication of UniPlate is cost‐effective, scalable, and compatible with 3D extrusion biopriniting techniques, which can be used to easily customize the tissue structures within the device. In this study, we demonstrated simple 3D printed tubular structures as a proof‐of‐concept. We showed that fibroblasts and pericytes can also be embedded inside the gel to form multilayered tubular blood vessels. In future studies, much more complex 3D tissue structures can be printed using gelatin, alginate, or Pluronic acid as sacrificial templates.^[^ ^24^, ^47^ ^]^ Parenchymal tissues, spheroids, aggregates, or organoids could also be embedded inside or placed on top of the gel to further increase model complexity.^[^ ^48^ ^]^ As stromal cells and parenchymal tissues are incorporated, future studies could explore the formation of oxygen and nutrient gradients that spontaneously arise from unidirectional perfusion. However, built‐in oxygen sensors may be required to detect such gradients directly. The body of the device was made of polystyrene using the injection molding method, which is consistent with the manufacture of industry‐standard microtiter plates. This device eliminates the nonselective drug absorption issue in PDMS devices, which can absorb up to 90% of hydrophobic molecules in solution.^[^ ^23^ ^]^

Although there are many benefits, the platform is not without limitations. The design principle demonstrated here can cause backflow in tubular tissues during the recirculation process when the rocker tilt direction changes from 15° to −40° tilt angle. However, the backflow process is minimal and will finish in seconds once the rocker returns to its original 15° tilt angle. Because the duration of backflow is 30 times smaller than that of forward perfusion, we assumed that the impact of backflow on cells is negligible. There are multiple potential factors that may cause the lack of endothelial cells alignments in 3D tubular tissues in our study, including the stiffness, shear stress, and interstitial flow in 3D gel matrix. The presence of a brief backflow could be another potential contributor, even though it lasts for only seconds. The precise control of the flow rate and shear stress is also difficult on this platform, particularly if a high flow rate (>250 µL min^−1^) and shear stress (> 2 dyne cm^−2^) are required. The wall shear stress is determined by a number of factors such as the diameter of the tubular tissue, porosity of the hydrogel, tilt angle of the programmable rocker, and media volume in the inlet and outlet wells. Some of these parameters, such as diameter and gel porosity, could be highly dynamic and subject to changes even during an experiment. Furthermore, as the media drains from the inlet to outlet wells, the flow rate and shear stress will inevitably change during the process. Therefore, we can only guarantee that the cultured tissues are exposed to shear stress and flow rate within a certain range but not of a precise value. In this study, the shear stress was adjusted by changing the tilt angle of the programmable rocker within a specified range. The accessible shear stress in this tubular tissue design is 0–2 dyne cm^−2^ which is applicable in a number of applications. For instance, this level of wall shear stress has been reported to facilitate vascular formation inside kidney organoids effectively.^[^ ^11^ ^]^

An important advancement of this platform is the continuous recirculation of circulating immune cells in engineered tubular tissue models. The circulating monocytes were maintained in suspension and recirculated. As they were perfused through engineered blood vessels, each monocyte would have passed through the blood vessels and interacted with the endothelium, on average, 250 times in 24 h. Previously, monocyte perfusion experiments were almost always performed by perfusing monocytes once through a tissue within a short period of time. Without sufficient exposure time, the extent of monocyte recruitment and the immune response can be severely underestimated. However, continuous recirculation allows monocytes and other immune cells to accumulate and interact with injured tissues, making it possible to model chronic tissue inflammation, repair, and regeneration.^[^ ^49^ ^]^ Monocytes and circulating immune cells are crucial components in immunological surveillance, and more accurately recapitulating monocyte recirculation in engineered tissues will provide better guidance in disease modeling, clinical disease treatment and drug discovery.^[^ ^50^ ^]^

## 4 Conclusions

In summary, we developed a pumpless recirculating platform that allows unidirectional flow through 3D printed vascular/tubular tissues. Single‐channel and duo‐channel designs were created to model blood vessels and vascularized proximal tubules with recirculating flow driven by gravity for the first time. Additionally, monocytes were successfully perfused and recirculated for at least 24 h without impairing cell viability or triggering inflammatory activation, indicating the potential of this platform for culturing circulating immune cells in a complex organ‐on‐a‐chip environment under unidirectional recirculating flow. This platform opens up possibilities for modeling and studying chronic inflammatory diseases by prolonging the interaction of circulating immune cells with endothelium and epithelial tissues. Ultimately, this improved our ability to better control the microenvironment in microphysiological systems for pathological modeling and drug discovery.

## 5 Experimental Section

### Unidirectional Plates Fabrication

All designs of customized bottomless wells and gelatin patterns were created using the AutoCAD software. The master molds for injection molding were printed using a Formlab printer (Formlabs, Form3B) following the supplier's instructions. Considering the high working temperature of the injection molding process, high‐temperature resin (Formlabs, cat. # RS‐F2‐HTAM‐02) was selected as the master mold. After printing, two post‐processing steps were performed to completely remove the noncrosslinked resin. Briefly, the print was immersed in isopropanol (ACS reagent, ≥99.5%, Sigma‐Aldrich, cat# 190764‐4L) and sonicated for 1 h. Next, the print was transferred to a UV chamber (Formlabs, FH‐CU‐01) and cured by UV exposure (light wavelength: 405 nm) for 2.5 h at 65 °C. The printed master molds were ready for use. A benchtop injection‐molding machine (Galomb, Model S‐100) was used to manufacture the customized bottomless wells (Figure S1, Supporting Information). The working temperature of the injection molder was set to 237 °C which is the maximum temperature that the heating unit allows. Ease release 205 (Sculpture Supply Canada) was applied to the master mold for the easy release of injection‐molded parts. Next, polystyrene resin pellets (Galomb, cat# RSN‐003‐1) with a melting point at 221–232 °C were loaded to the cartridge. Once the polystyrene pellets became molten plastic in ≈3 min, the handle was manually pulled down. In this process, molten polystyrene is pressed into master molds. After pressing for 20 s, the parts were released from the mold. The plastic parts were then soaked in isopropanol for 1 h to removing the residual release reagent on the surface.

To fabricate the base of the device, 40% w/v gelatin (Sigma‐Aldrich, cat# G9391‐100G) was printed on a pressure‐sensitive adhesive sheet (Strouse, cat# 3M‐9795R) using an extrusion‐based bioprinter (Cellink, BIO X). The commercially available pressure‐sensitive adhesive (PSA) tape 3M‐9795R was selected because of its nontoxicity toward cells, excellent sealing capability and bioassay compatibility. The 3M‐9795R tape is designed as a microfluidic diagnostic adhesive tape. As the 3M‐9795R tape shows a chemically inert surface, consisting of a clear polypropylene film coated with a pressure‐sensitive silicone adhesive, it has been applied in microfluidic lab‐on‐chip systems for cell culture, tumor angiogenesis, and  polymerase chain reaction studies.^[^ ^51^, ^52^, ^53^ ^]^ It has also been reported that the maximum bursting pressures for PMMA, polycarbonate, and 3D printing resin adhered to PSA were above 5 bar at room temperature, comparable to the bond strengths generated by plasma treatment in PDMS‐PDMS and PDMS/glass, which typically range from 1 to 5 bar.^[^ ^54^ ^]^ Moreover, the cytotoxicity of PSA has been evaluated by culturing MDCK epithelial cells and developing tumor angiogenesis models, with results showing no apparent differences in cell adhesion, cell growth, and cell spreading compared to glass.^[^ ^25^ ^]^ The bioprinter was set to work under a working pressure of 15 KPa and printing speed of 5 mm s^−1^ using a 27‐gauge (inner diameter: 0.2 mm) needle. During the gelatin printing, the temperature of gelatin cartridge was maintained at 50 °C, while the print bed temperature was set to 12–14 °C which helps to set gelatin instantly. With the bioprinter, gelatin fibers with around 200 µm diameter were patterned on the adhesive sheets. The adhesive sheets were air‐dried for an hour after gelatin printing, which allows us to bond bottomless wells to pressure sensitive adhesive sheets under a dry condition effectively. The dried gelatin template works well as the sacrificial scaffold in forming simple single tubular channels with a uniform shape (Figure S3, Supporting Information). Before combining the adhesive sheet with the injection‐molded bottomless wells, the bottom side of the bottomless well was treated with a corona treater (Electro‐Technic Products Inc., Model BD‐20) for 2 min. The pressure‐sensitive adhesive sheet with printed gelatin was hand‐pressed onto bottomless wells. The pressure‐sensitive adhesive becomes sticky only in the region that is exposed to compression forces. Therefore, this material does not affect the area inside the wells.

The entire process was repeated eight times to create eight devices. The devices were then assembled in a OneWell Plate (VWR, cat# 30617‐434) and secured in place using a 3D printed frame. The frame was printed using a FormLab printer. Then, one full UniPlate device was packaged in a sealing bag and stored at room temperature until use. All plates were sterilized using an ultraviolet chamber (Benchmark Scientific, UV Clave) for 20 min before use. All the UniPlate devices were used only once.

### Drug Absorption of PSA

Rhodamine B was used for determining absorption of PSA to small hydrophobic molecules. Standard 384‐well plates were selected as the polystyrene samples. PSA samples were prepared by assembling PSA sheet with a bottomless 384‐well plate, and the PDMS samples were prepared by casting a PDMS (1:10) layer on polystyrene samples. Then, each sample was incubated with 2 µM Rhodamine B (Sigma‐Aldrich, cat# 81‐88‐9) solution at 37 °C overnight, followed by three times of phosphate‐buffered saline (PBS) wash. After washing, fluorescent images of samples were captured by the plate reader.

### Quantification of Flow Rate and Shear Stress

To calculate the volumetric flow rates (Q) through the blood vessel model, 1 mL and 50 µL of PBS was added to the inlet and outlet wells, respectively. Then the plate was placed on a rocker tilted at an angle (10^○^, 15^○^, 20^○^, 24^○^, and 30^○^). After 5 min, the liquid accumulated in the outlet wells were collected, and the liquid volume was measured. To understand flow rate changes in one perfusion cycle at 15^○^ tilt angle, 1 mL and 300 µL of PBS was added to the inlet and middle wells, respectively. Then, the liquid accumulated in outlet wells were collected every 30 s and liquid volumes were measured. The flow rate of backflow in the recirculation step was determined by collecting liquid that flowed from outlet walls to inlet walls through blood vessels in 20 s (Figure S9c, Supporting Information). Wall shear stress (τ) was calculated using Equation (1)

(1) $$\tau \;=\frac{4Q\times \eta }{\pi r^{3}}\;$$

The diameter of the tubular vessel was measured from brightfield images using ImageJ, assuming that the vessel has a circular cross‐section.

### Cell Culture

Green fluorescent protein‐expressing human umbilical vein endothelial cells (GFP‐HUVECs, Angio‐Proteomie, cat# CAP‐0001GFP) were cultured in Endothelial Cell Growth Medium 2 (ECGM2, Sigma‐Aldrich, cat# C‐22011) according to the manufacturer's protocol. Human renal proximal tubular epithelial cells (RPTEC‐TERT1, Everycyte, cat# CHT‐003‐0002) were cultured in ready‐to‐use ProxUp medium (Evercyte, cat# MHT‐003). Human lung fibroblasts were cultured in Dulbecco's Modified Eagle Medium (DMEM, Thermo Fisher Scientific, cat# 11 995 065) supplemented with 10% fetal bovine serum (FBS, Thermo Fisher Scientific, cat# 12 484 028). Human pericytes from placenta (hPC‐PL, Sigma‐Aldrich, cat# C‐12980) were cultured in pericytes growth medium 2 (Sigma‐Aldrich, cat# C‐28041) according to the manufacturer's protocol. THP‐1 monocyte cells were cultured in RPMI 1640 medium (Cedarlane Labs, cat# 30‐2001) supplemented with 10% FBS and 2‐mercaptoethanol (0.05 mM) in nontreated multiple well plates. To prepare for further experiments, cells were plated in T75 cell culture flasks and grown in an incubator with 5% CO_2_ at 37 °C. The endothelial cells used in this study were between passages 3–6. Proximal tubule epithelial cells were cultured until passage eight.

### 3D Tissues Formation and Plates Operation

Fibrin gel was prepared by mixing fibrinogen (Sigma Aldrich, Cat# F3879‐1G) and thrombin (Sigma‐Aldrich Cat#, T6884‐100UN). A stock solution of thrombin was prepared at a concentration of 10 U mL^−1^ in 0.1% BSA (Sigma‐Aldrich, Cat# A9205‐10ML). Fibrin gel was prepared by mixing 100 µL of fibrinogen (10 mg mL^−1^) and 20 µL of thrombin (10 U mL^−1^) and then immediately cast into the tissue wells to encapsulate the gelatin template. After gelation at room temperature for 15 min, the plate was transferred to the incubator and incubated with PBS overnight at 37 °C. As gelatin has a melting point in the range of 31.7–34.2 °C, gelatin will melt and dissolve in PBS in the incubator. Thus, an open lumen was generated inside the hydrogel after aspirating the gelatin dissolved in PBS, followed by rinsing with the complete medium. To prevent fibrin gel degradation over time, all cell culture media were supplemented with 1% v/v aprotinin (Sigma‐Aldrich, Cat# 616370‐100MG‐M). The cell culture medium was changed daily.

To compare differences of printed blood vessels cultured under static, bidirectional, and unidirectional conditions, cell morphology, cell orientation, and cytokine secretion levels were analyzed. The shape index and orientation of endothelial cell nuclei in confluent blood vessels after 7 days of culture were measured. The media perfusates from blood vessels cultured on static, bidirectional, and unidirectional conditions on day 8 were collected, and cytokine analysis was performed to quantify cytokine secretion levels in each group.

For cell seeding, 200 µL of cell suspension (2 million cells mL^−1^) was added in both inlet and outlet wells, where the cells will flow into the tubular channels in fibrin gel. The plate was placed on a level surface in an incubator under static condition to allow cells attachment for 1 h. After 1 h, the plate was moved to a rocker to initiate unidirectional or bidirectional perfusion. ECGM2 medium with 1% aprotinin was used to culture the vascular tissues. For the duo‐channel design, endothelial cells and renal proximal tubule epithelial cells were seeded at the same time. Generally, 200 µL of endothelial cells suspension and 200 µL of RTPECs suspension were added in their corresponding inlet and outlet wells. ProxUp medium containing 1% aprotinin was used for the proximal tubule until it reached 100% confluency. The medium was then changed to a modified DMEM medium (no glucose, Thermo Fisher Scientific, Cat# 11 966 025) supplemented with 100 mg dL^−1^ D‐(+)‐glucose (Sigma‐Aldrich, Cat# G5767‐500G) and ProxUp supplements (Cat# MHT‐003‐S).

### Permeability Assay

The barrier function of the tubular blood vessel model was measured by perfusing a TRITC‐labeled dextran solution (65 kDa, Sigma‐Aldrich, cat# T1162) through the vessel. Specifically, 300 and 100 µL of culture media were added to the tissue well and outlet well, respectively, and 500 µL of TRITC‐labeled dextran (400 µg mL^−1^) solution was added in the inlet well. Time‐lapse images were captured using a microplate reader (BioTek, Cytation 5) at 15 min intervals. From the time‐lapse images, the permeability and *P*_d_ for each vascular tissue were calculated using Equation (2)

(2) $$\;P_{d}=\frac{d}{4}\times \frac{1}{\Delta t}\left(\frac{I_{f}-I_{i}}{I_{i}-I_{b}}\right)$$

In Equation (2), *I*_f_ and *I*_i_ represent average intensities at the final and initial time point, while *I*_b_ represents the average intensity for background. Δ*t* is the time interval between images, and *d* is average diameter of vascular tissues.

### Glucose Transport Studies

For the glucose transport experiment, ECGM2 media and modified DMEM were mixed at a 1:1 v/v ratio and then added to both vascular and proximal tubule inlet/outlet wells on day 7 after the proximal tubule reached full confluency. Media perfusates from the proximal tubule and vasculature outlets were collected after 24 h of perfusion. Glucose levels in all collected perfusates were measured using a glucose meter (Contour Next One Meter).

### Monocytes Recirculation and Cytokines Analysis

To demonstrate the feasibility of recirculating suspended cells in UniPlate, 700 µL of human monocytes (THP‐1) suspension (1.5 million cells mL^−1^) was added in the inlet well and 200 µL of culture media were added to the tissue and outlet wells. For the static condition, the plate was kept leveled in an incubator. For the recirculating condition, the plate was placed on the rocker. After 24 h, the culture media and circulating cells from both static and recirculating conditions were collected. To evaluate cell loss, cell numbers in both the inlet and outlet wells were counted using a hemocytometer. To evaluate the impact of mechanical stress from recirculation, the culture media was analyzed for inflammatory cytokines. Cytokine analysis was performed by Eve Technologies using the Human Cytokine Array Proinflammatory Focused 15‐plex panel (cat# HDF15).

### Immunostaining and Live/Dead Staining

Before fixation, all tissues were washed three times with fresh culture media to remove dead cell debris. The tissues were then fixed with 4% w/v paraformaldehyde solution for 20 min on a rocker at room temperature. Next, the tissues were washed with PBS buffer three times and blocked using a blocking solution (10% FBS in PBS) for an hour on a rocker at room temperature. After removing the blocking solution, tissues were incubated with primary antibodies, anti‐CD31 (Abcam, cat# ab9498), anti‐VE‐Cadherin (Abcam, cat# ab33168), anti‐Sodium Potassium ATPase (Abcam, cat# ab76020), or anti‐SGLT2 (Abcam, cat# ab85626) overnight at 4 °C. A blocking buffer containing 0.1% Triton X‐100 was used to stain VE‐cadherin, Na/K ATPase, and SGLT2. The tissues were washed with PBS three times to remove residual primary antibodies, followed by incubation with fluorochrome‐conjugated secondary antibodies, F‐actin (Cedarlane Labs, cat# 20553‐300) and DAPI (Sigma‐Aldrich, cat# D9542) for 1 h at room temperature. All primary and secondary antibodies were diluted in PBS containing 2% FBS. The tissues were washed again with PBS three times prior to imaging using a confocal microscope (ZEISS, 3i Marianas LightSheet). Live/dead staining was performed using 5‐Carboxyfluorescein diacetate (CFDA, Sigma‐Aldrich, cat# C4916‐25MG) and propidium iodide solution (PI, Sigma‐Aldrich cat# P4864‐10ML), following the manufacturer's instructions.

### Data Quantification and Statistical Analysis

The elongation and orientation of endothelial cells nuclei were determined by analyzing the circularity and orientation angle using particles analysis function from ImageJ. SigmaPlot software was used for testing the normality and equality of variance. One‐way ANOVA or Kruskal–Wallis one‐way ANOVA on ranks in conjugation with the Holm–Sidak method were used for determining statistical significance. Data in all graphs are plotted as mean with standard deviation (SD) using Prism 9 (GraphPad, USA), and at least three independent samples (*n* ≥ 3) were used in each condition to perform quantitative analysis.

## Author Contributions

F.Z. designed and fabricated the plates, performed the experiments, analyzed the results, and prepared the manuscript. D.S.Y.L. contributed to particle perfusion and monocyte perfusion experiments. S.R. contributed to renal proximal tubule cell culture and helped with cytokines analysis. A.S. optimized bioprinter parameters and helped with gelatin printing on pressure sensitive adhesive sheets. B.Z. envisioned the concept, supervised the work, and edited the manuscript.

## Conflict of Interest

The authors declare no conflict of interest.

## Supporting information

Supporting Information

Video 1

Video 2

Video 3

## Data Availability

The data that support the findings of this study are available from the corresponding author upon reasonable request.
