# Supplementary material for: Pump‐Less Platform Enables Long‐Term Recirculating Perfusion of 3D Printed Tubular Tissues
Source: Adv Healthc Mater. 2023 Sep 19;12(27):2300423. doi: 10.1002/adhm.202300423 (PMC11469154; doi:10.1002/adhm.202300423)
Supplement: Supplementary file 1 — Supporting Information [file ADHM-12-2300423-s003.pdf]

ADVANCED HEALTHCARE MATERIALS

Supporting Information

for *Adv. Healthcare Mater.*, DOI 10.1002/adhm.202300423

Pump-Less Platform Enables Long-Term Recirculating Perfusion of 3D Printed Tubular Tissues

*Feng Zhang, Dawn S. Y. Lin, Shravanthi Rajasekar, Alexander Sotra and Boyang Zhang**

Supporting information

Pump-less platform enables long-term recirculating perfusion of 3D printed tubular tissues

Feng Zhang[1], *Dawn S. Y. Lin*[2], *Shravanthi Rajasekar*[2], *Alexander Sotra*[1], *Boyang Zhang*[1,2]*

[1] School of Biomedical Engineering, McMaster University, Hamilton, Ontario L8S 4L8, Canada;

[2] Department of Chemical Engineering, McMaster University, Hamilton, Ontario L8S 4L8, Canada;

* Corresponding author, orcid.org/0000- 0002-2060-5555; Email: zhangb97@mcmaster.ca

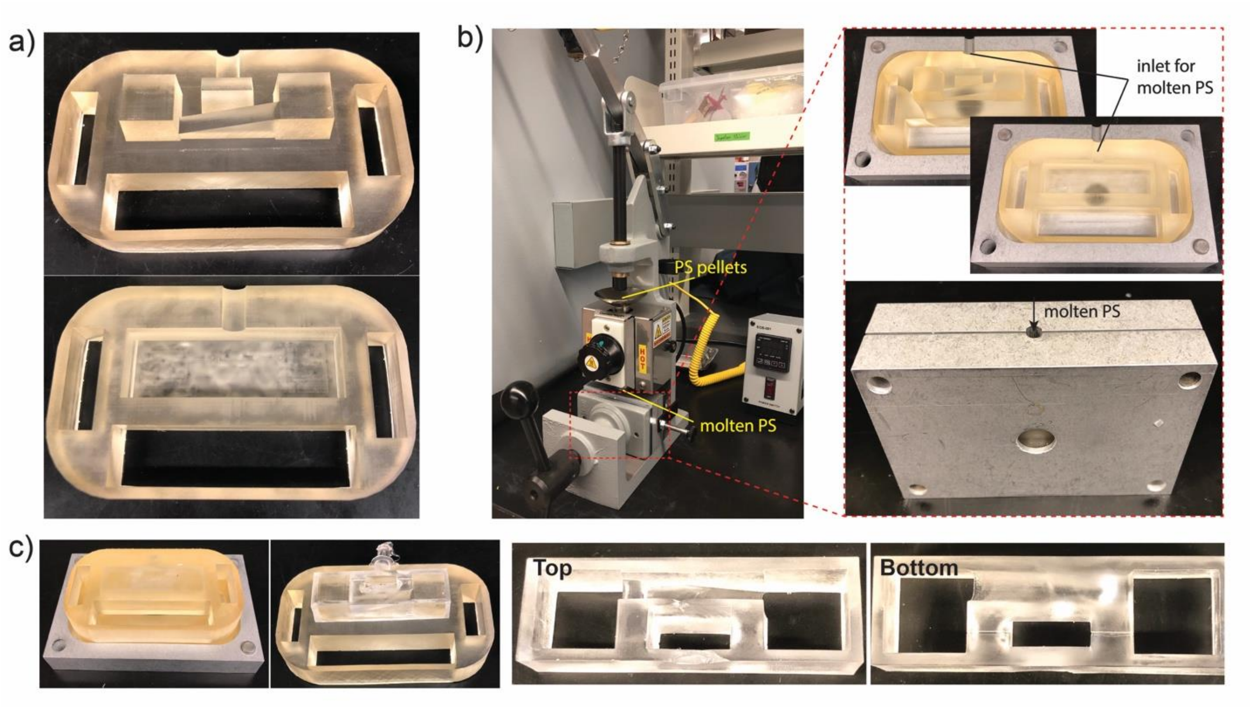

Supplementary Figure 1. Injection molding. a) 3D printed master molds for manufacturing customized bottomless wells with injection molding. The master molds are designed into two pieces for easy release. One piece of master mold contains the inner features of the customized bottomless wells, and the other defines the outer frame. b) Photos of desktop injection molder and setup. The 3D printed master molds are designed to fit into a pair of aluminum molds. c) The photos of manufactured bottomless wells before and after release from printed master molds.

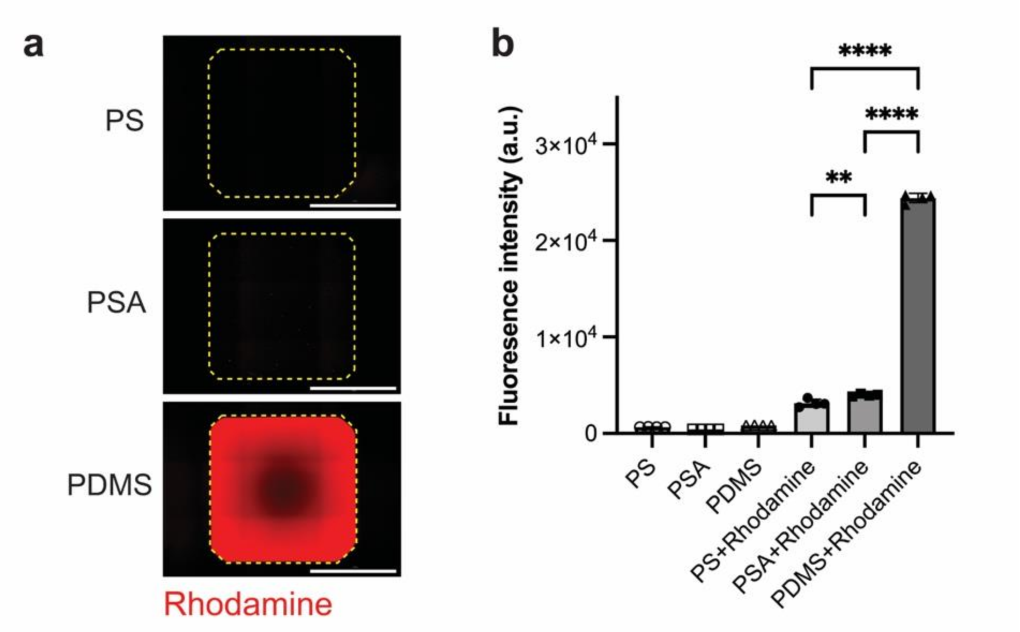

Supplementary Figure 2. Evaluation of drug absorption of pressure sensitive adhesive sheets to small hydrophobic molecule, Rhodamine B. Three types of samples, polystyrene (PS), pressure-sensitive adhesive (PSA), and PDMS were tested. Scale bar: 2 mm. n=4 samples for each condition. Statistics significance was determined using one-way ANOVA with the Holm–Sidak method. $^{**}P < 0.01$, $^{****}P < 0.0001$.

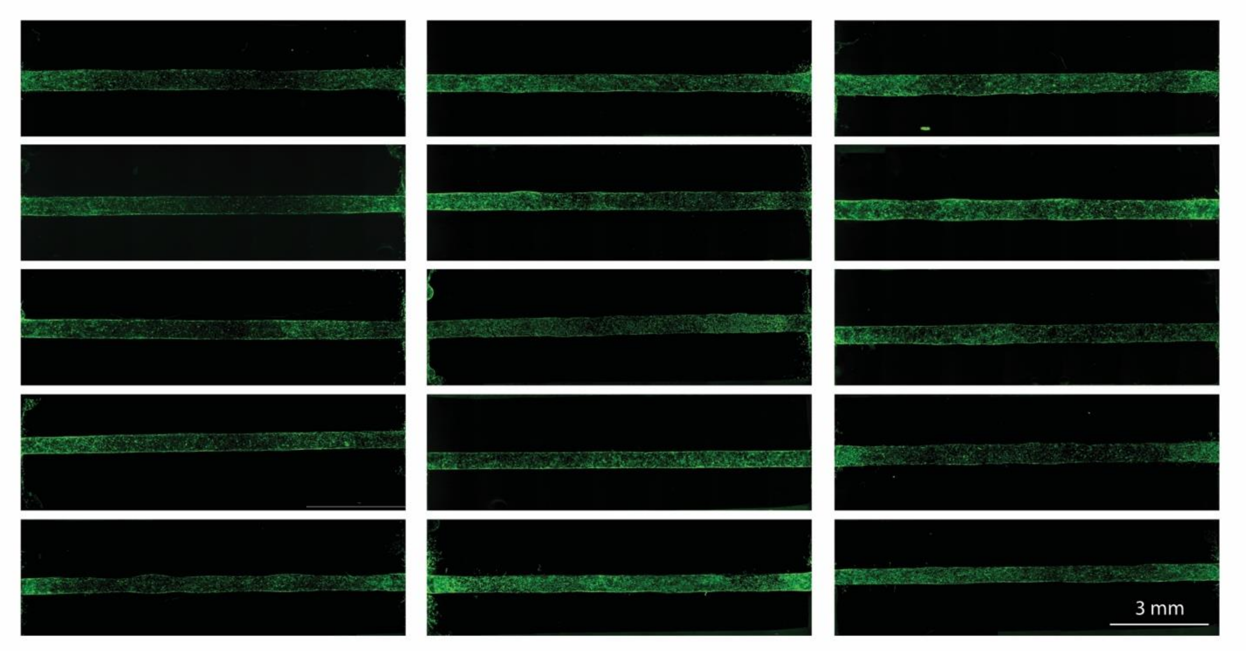

Supplementary Figure 3. Image of 15 representative single-channel blood vessels seeded with GFP-HUVECs.

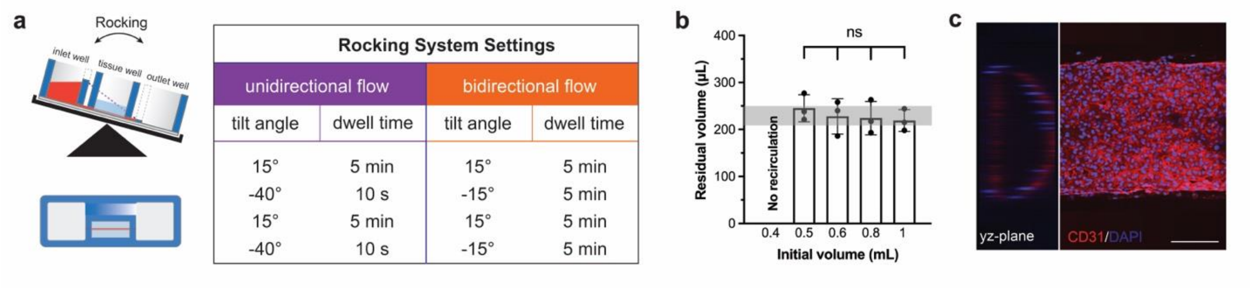

Supplementary Figure 4. a) The setting parameters of programable rocker for bidirectional and unidirectional flow conditions. b) Correlation of residual volume in UniPlate under various initial liquid volumes added in outlet wells at a -30º tilt angle during the recirculation process; n=3 devices for each condition. Statistics significance was determined using one-way ANOVA. “ns” indicates not statistically significant. c) Confocal projected z-stack fluorescence images of blood vessels with bidirectional flow culture; Cells were stained for CD31 (red) and DAPI (blue). Scale bar: 200 µm.

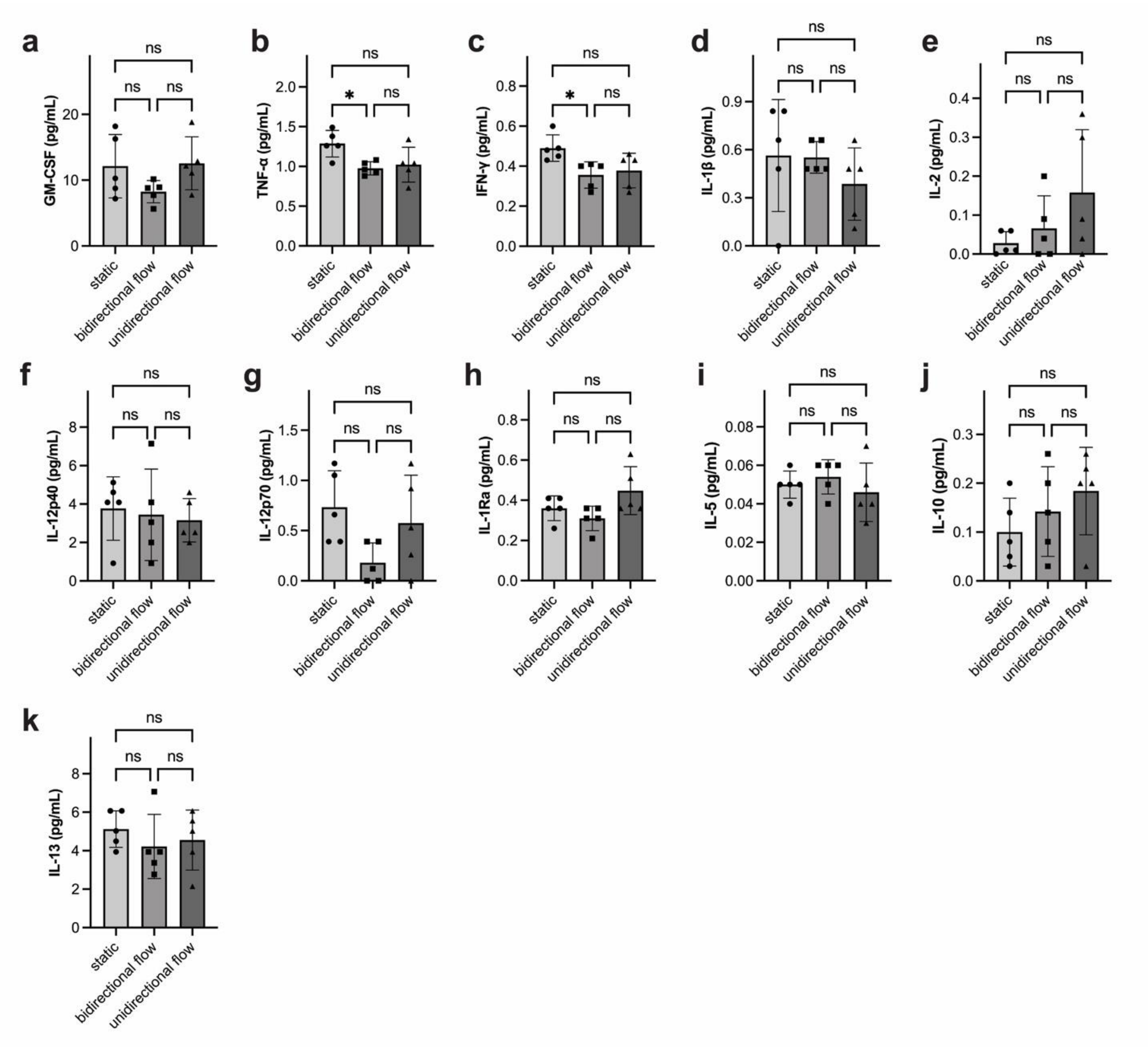

Supplementary Figure 5. Secretion levels of all analyzed inflammatory-related cytokines, including : GM-CSF (a), TNF-α (b), IFN-γ (c), IL-1β (d), IL-2 (e), IL-12p40 (f), IL-12p70 (g), IL-1Ra (h), IL-5 (i), IL-10 (j), IL-13 (k) in collected media perfusates from blood vessels cultured under static, bidirectional flow and unidirectional flow conditions. n=5 for each condition. Statistical significance was determined using one-way ANOVA with the Holm–Sidak method. *$P < 0.05$, “ns” indicates not statistically significant.

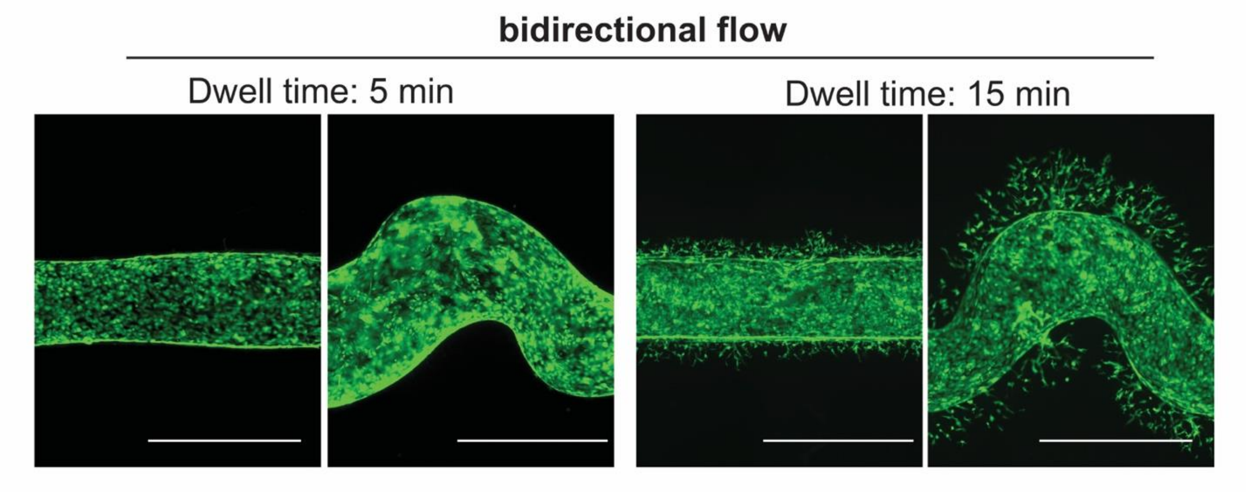

Supplementary Figure 6. GFP-expressed endothelial cells-lined vessels formed after 7 days of culture under bidirectional flows with 5 min or 15 min dwell time; Scale bar: 1 mm.

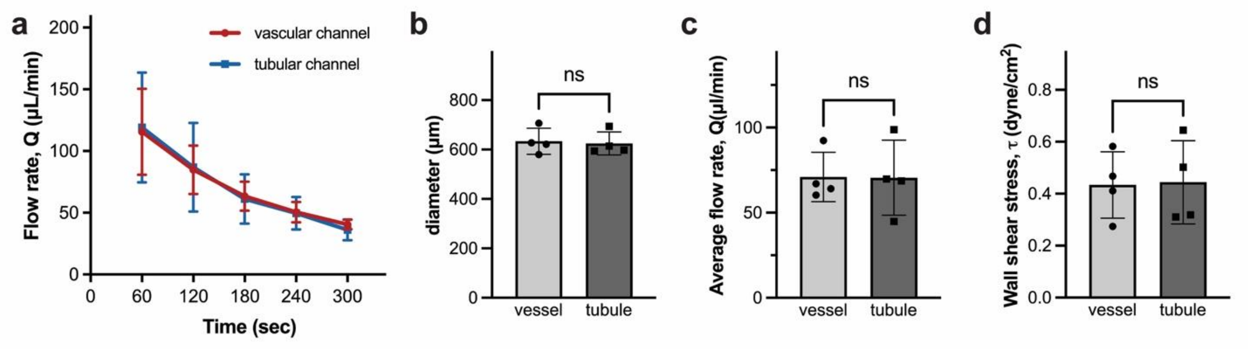

Supplementary Figure 7. Flow quantifications for the duo-channel UniPlate in one perfusion cycle at 15º tilt angle. a) Flow rate changes over time in vascular and tubular channels in duo-channel UniPlate at 15º tilt angle. Flow rate was measured every minute. n=4 devices. b) Average diameter of vascular and tubular channels. n=4 devices for each condition. c) Average flow rate in vascular and tubular channels in 5min perfusion at 15º tilt angle. n=4 devices for each condition. d) Average wall shear stress in both vascular and tubular channels at 15º tilt angle. n=4 devices for each condition. Statistics significance was determined using one-way ANOVA. “ns” indicates not statistically significant.

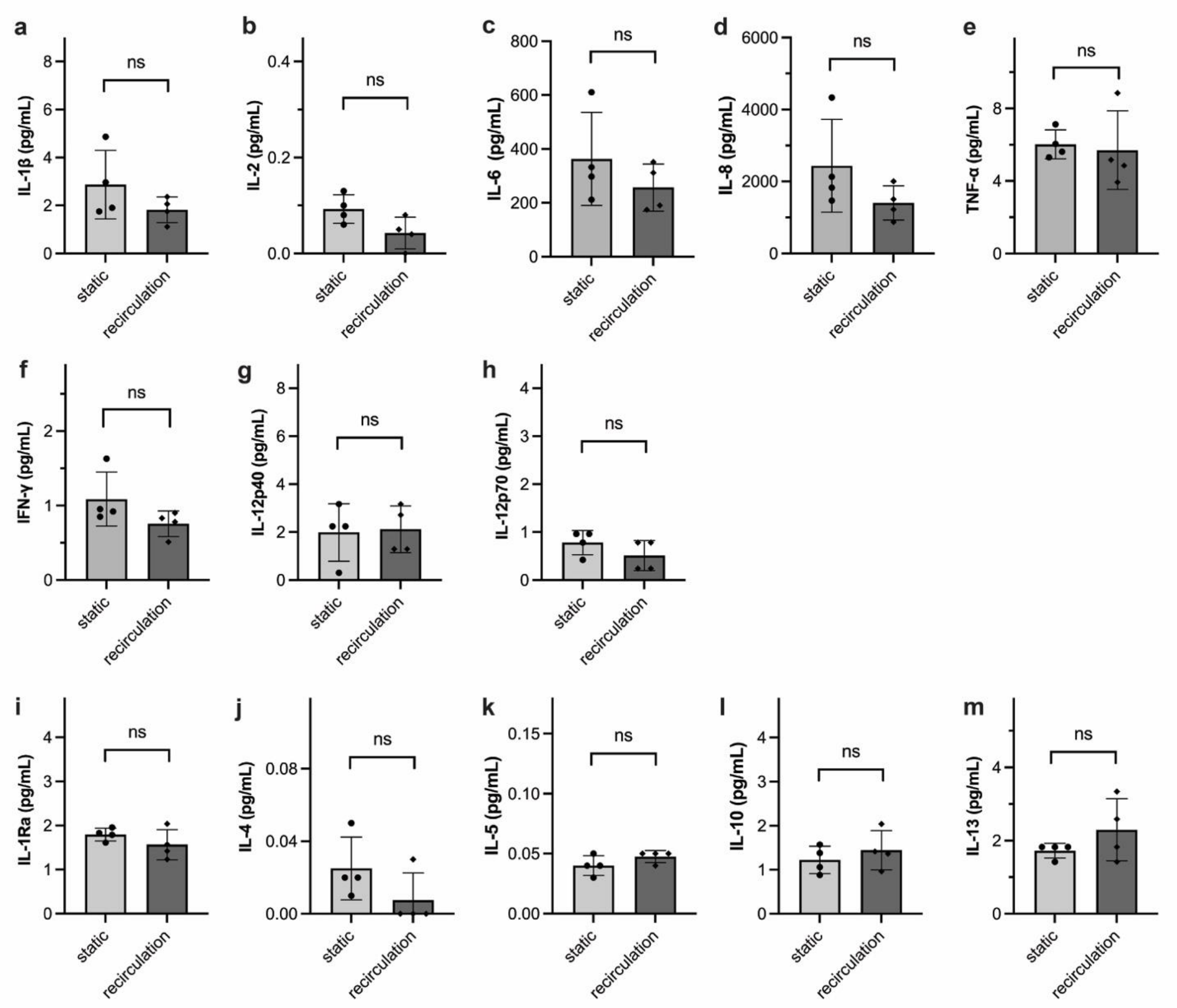

Supplementary Figure 8. Secretion levels of all analyzed inflammatory-related cytokines, including: IL-1β (a), IL-2 (b), IL-6 (c), IL-8 (d), TNF-α (e), IFN-γ (f), IL-12p40 (g), IL-12p70 (h), IL-1Ra (i), IL-4 (j), IL-5 (k), IL-10 (l), IL-13 (m) in static and recirculation groups. n=4 devices for each condition. Statistics significance was determined using one-way ANOVA. “ns” indicates not statistically significant.

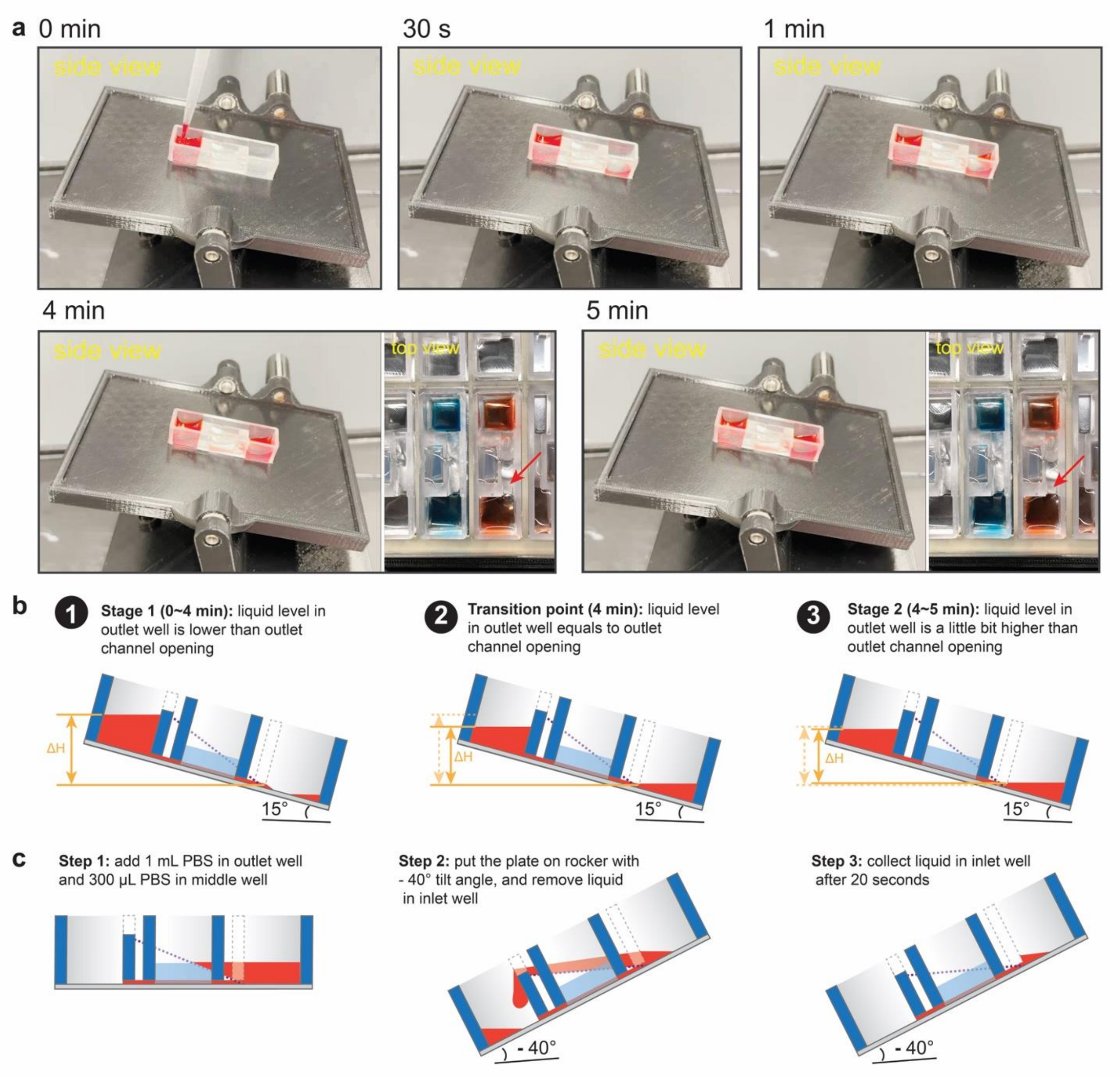

Supplementary Figure 9. a) Time frame images of liquid perfusion in one recirculation cycle. In the images of 30 s and 1 min time points, the liquid accumulated in outlet well gradually distributed to all corners of the outlet well, but the liquid level in outlet wells is lower than the outlet channel opening. In the images of 4 min and 5 min time points, the liquid level remained comparable to outlet channel opening. The red arrow shows the clean and dry bridge edge near the outlet well. b) Schematic images for showing changes of liquid level differences in inlet and

outlet wells with time. c) Schematics of setup and process for evaluating the flow rate of backflow.

Supplementary Video 1: Unidirectional perfusion and recirculation in UniPlate.

Supplementary Video 2: Particle perfusion in tubular blood vessel in UniPlate to show unidirectional flow and the transient backflow in one perfusion cycle.

Supplementary Video 3: Monocytes perfusion in a tubular blood vessel in UniPlate.
